# Negative predictive values for three main groups of medicaments for quickly resolve food allergic reactions in children

**DOI:** 10.1186/2045-7022-1-S1-P8

**Published:** 2011-08-12

**Authors:** Adnan Bajraktarevic, Andrea Pahor Kurilic, Sanja Putica, Begeler Begovic, Amina Selimovic, Asmir Musabegovic, Teodora Frankic, Haris Niksic, Nermina Korac, Aida Djulepa Djurdjevic, Branka Djukic, Lutvo Sporpsevic

**Affiliations:** 1 Public Health Institution of Canton Sarajevo, Pediatrics Department, Sarajevo, Bosnia and Herzegovina; 2 Clinical Medical Center Sarajevo, Clinical Pharmacology, Sarajevo, Bosnia and Herzegovina; 3 Pediatrics Clinic Sarajevo, Department for allergology and pulmonology, Sarajevo, Bosnia and Herzegovina; 4 Pharmacy Faculty Sarajevo, Clinical Pharmacology, Sarajevo, Bosnia and Herzegovina; 5 General Hospital Sarajevo, Emergency Department, Sarajevo, Bosnia and Herzegovina; 6 First Medical Aid New Sarajevo, Pediatrics Department, Sarajevo, Bosnia and Herzegovina

## Background

The pediatrician plays an important role in contributing to the management of children with food allergies. Antihistamine drugs are used to control or alleviate the allergy symptoms like, skin rash or hives and breathing difficulties, by counteracting the effects of histamine. Dexamethasone is a potent corticosteroid and it acts as an anti-inflammatory and immunosuppressant. Adrenaline or epinephrine is the drug of choice for treating anaphylaxis.

## Methods

We describe the recruitment of 1000 children for participation in a randomized trial examining the effectiveness of strategies for the management of pediatric food allergy reactions for shorter time maximal up to five days during period 2000-2010. Depends from severity of allergy and level of allergic reactions , authors used first oral antihistamines, loratidine, second intramuscular corticosteroid, dexamethasone and third intravenose catecholamine , epinephrine in doses for rules of allergy reactions by food in children.

## Results

Staistics calculation and negative predictive values for three main groups of medicaments for quickly resolve food allergic reactions in children showed that loratidine has had no success in 16% cases, after that dexamethasone in following therapy for only about 2.5 % cases, and the end as third adrenaline for 0.3% cases in children age 0 to 19. Reported food allergy is increasing among children of all ages, among boys and girls, and among children of different Bosnian ethnicities. There were no differencies in responding on therapy of this three groups of medicament related on sex and members for region, but significant bad response on therapy on Roma children population.

## Conclusions

The most significant reactions in children are attributable to industrial meat as sausages and hot dogs, egg, strawberries, peanut, tree nuts ( walnut, etc), milk, fish, shellfish, citrus fruits, herbs, soy, tomato and wheat. Longitudinal follow-up of the effect of antiallergic therapy on children and satisfaction with therapy of these parents and their families is currently underway.

## Key Words

Food Allergy, Children, Antihistamines, Corticosteroids, Epinephrin

